# Apical hypercontractility mitigates impaired diastolic filling and lower intraventricular haemodynamic forces in human bed rest

**DOI:** 10.1113/EP093671

**Published:** 2026-06-29

**Authors:** Jérémy Rabineau, Fatimah Al‐Darwish, Edwin Mulder, Bram F. Coolen, Fabian Hoffmann, David Hautemann, Jens Tank, Pierre‐François Migeotte, Gustav J. Strijkers, Rob C. I. Wüst

**Affiliations:** ^1^ LPHYS, Department of Cardiology Université Libre de Bruxelles Bruxelles Belgium; ^2^ Department of Kinesiology and Health Sciences University of Waterloo Waterloo Ontario Canada; ^3^ Department of Biomedical Engineering and Physics, Amsterdam Cardiovascular Sciences, Amsterdam UMC University of Amsterdam Amsterdam The Netherlands; ^4^ Institute of Aerospace Medicine German Aerospace Center Cologne Germany; ^5^ MEDIS Leiden The Netherlands; ^6^ Department of Human Movement Sciences, Faculty of Behavioural and Movement Sciences, Amsterdam Movement Sciences Vrije Universiteit Amsterdam The Netherlands

**Keywords:** cardiac contractility, haemodynamic forces, inward displacement, non‐invasive imaging, physical inactivity

## Abstract

Prolonged physical inactivity alters the cardiovascular system, including the heart. Long‐term bed rest is known to decrease left ventricular volume and cause diastolic dysfunction; however, the interplay between these changes and their effect on cardiac contractility have been understudied. Here we used novel non‐invasive imaging techniques to longitudinally assess changes in regional cardiac contractility and function that can occur prior to the development of overt cardiac changes. Specifically, we performed cardiac magnetic resonance imaging on 24 healthy participants before, during, and after 60 days of strict head‐down tilt bed rest (AGBRESA) to measure intracardiac haemodynamic forces and left‐ventricular inward displacements via dedicated software. The integrated left ventricular haemodynamic force as well as the ejection force during systole were reduced throughout the bed rest. While maximal diastolic deceleration (at the E‐wave) significantly decreased during bed rest, left ventricular diastolic suction and maximal value for the late diastolic deceleration phase were significantly increased. Inward displacement analysis showed that motion of the cardiac base was significantly reduced after bed rest, whereas apical motion was significantly increased. The analysis of haemodynamic forces and regional cardiac inward displacement are novel non‐invasive imaging readouts that can provide longitudinal information about cardiac contractility and function. Our study highlights that prolonged bed rest alters regional cardiac motion and haemodynamic forces, with a desynchrony in contractile alterations to maintain cardiac filling during diastole.

## Abstract (highlights)

- **What is the central question of this study?**
  How does prolonged strict bed rest alter intraventricular haemodynamic forces and regional left ventricular wall motion in healthy humans, and can advanced non‐invasive imaging detect early, subclinical changes in cardiac pump mechanics during bed rest?
- **What is the main finding and its importance?**
  Sixty days of bed rest reduced global haemodynamic force production in healthy humans, while early diastolic suction increased, accompanied by apical hypercontractility. These findings reveal region‐specific mechanical adaptations that help preserve cardiac filling during deconditioning, demonstrating that novel non‐invasive imaging techniques detect subtle cardiac alterations beyond conventional measures.

## 1 INTRODUCTION

A sedentary lifestyle or forced immobility in hospitalized patients is associated with increased cardiovascular risks and mortality (Fletcher et al., 2018). The cardiovascular system in (simulated) microgravity and bed rest also suffers from hypokinesia and a whole‐body headwards fluid shift. A reduction in plasma volume occurs within the first few days and can reach more than 10% after long‐term bed rest in humans (Jirak et al., 2022; Johansen et al., 1997; Levine et al., 2022). Physical inactivity – even for a period as short as 2 weeks – reduces left ventricular (LV) volume and mass, and causes diastolic dysfunction (Dorfman et al., 2008; Hoffmann et al., 2021; Perhonen et al., 2001). Besides this, stroke volume and cardiac strain decrease after long‐term exposure to (simulated) microgravity (Hoffmann et al., 2021).

It is highly desirable to have non‐invasive methods that can measure the early subtle changes in myocardial function caused by environmental stressors. This can help us gain a better understanding of the progression of healthy cardiac function toward heart failure, whether with or without preserved ejection fraction, and aid in the development of new therapeutic interventions. Magnetic resonance imaging (MRI) has emerged as a versatile translational imaging modality, and exciting new advancements allow for the quantitative assessment of cardiac function that goes beyond simply measuring ejection fraction (Daal et al., 2022; Monosilio et al., 2025; Wüst et al., 2019, 2021). A novel imaging biomarker is the assessment of cardiac haemodynamic forces, which provides an integral evaluation of myocardial function by directly evaluating the efficiency of cardiac ejection and filling (Faganello et al., 2020; Lapinskas et al., 2019; Vallelonga et al., 2021; Vos et al., 2022), taking into account alterations in stroke and LV volume. Preclinical and cross‐sectional human studies revealed that cardiac haemodynamic forces are greater in athletes and lower in sedentary patients with heart failure (Arvidsson et al., 2017, 2018; Eriksson et al., 2016; Ferrara et al., 2021; Laenens et al., 2023; Lapinskas et al., 2019; Monosilio et al., 2025; Vos et al., 2022), but longitudinal data on how environmental stressors, such as physical inactivity, affect intraventricular pressure gradients are lacking.

Another promising non‐invasive imaging biomarker that can detect subtle differences in cardiac contractility is the assessment of LV inward displacement. This technique measures the LV wall motion toward the midline of the LV, providing valuable insights into cardiac performance, allowing assessment of segmental wall motion abnormalities, dyskinesia, or hypercontractility (de la Pena‐Almaguer et al., 2021). This imaging biomarker provides additional information on cardiac contractility in patients with ischaemic heart failure and reduced ejection fraction who have undergone minimally invasive LV reconstruction (Hegeman et al., 2023b). At present, it remains unclear how regional cardiac contractility and wall motion are linked to whole cardiac function, and whether bed rest results in longitudinal alterations in regional wall motion in humans.

The objective of this study was therefore to evaluate intraventricular haemodynamic forces and LV inward displacement using MRI in 24 healthy humans before, during, and after a 60‐day period of strict head‐down‐tilt bed rest.

## 2 METHODS

### 2.1 Ethical approval

The research was part of the 60‐day strict head‐down tilt bed rest study ‘Artificial Gravity Bed Rest with European Space Agency’ (AGBRESA) organized by the American and European space agencies, as well as the German Aerospace Center (NASA, ESA, and DLR, respectively). The AGBRESA study was compliant with the *Declaration of Helsinki*. It was approved by the Northern Rhine Medical Association (Ärztekammer Nordrhein, no. 2018143) and the Federal Office for Radiation Protection (Bundesamt für Strahlenschutz, no. 22464/2018‐074‐R‐G), and it was registered in the German Clinical Trials Register (DRKS00015677). Written informed consent was obtained from all the participants.

### 2.2 Study design and population

**FIGURE A1:**
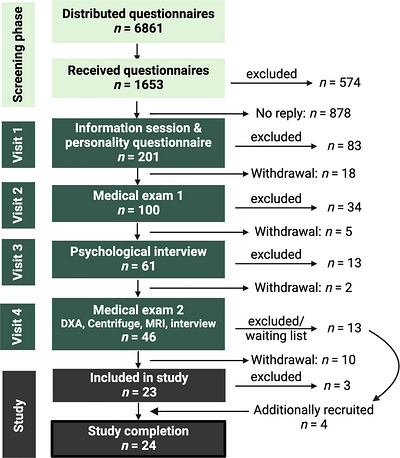
Participant screening process. Out of 6861 distributed questionnaires, 1653 potential participants received additional information about the study, and 201 people joined an on‐site visit and assessment at the German Aerospace Centre. Three subsequent on‐site visits included more rigorous assessments, and 23 healthy participants were initially included in the study. Three participants were excluded and replaced by four participants on the reserve list.

Appendix Figure A1 provides a CONSORT diagram of the inclusion of the study participants. Twenty‐four healthy study participants (8 females, age range: 24–54 years, BMI: 24.3 ± 2.0 kg m^−2^) stayed 88 consecutive days at the aerospace medicine research facility :envihab (facility in Cologne, Germany). Their stay included: 14 days of baseline data collection, 60 days of head‐down tilt bed rest, and 14 days of supervised recovery. During the bed rest period, the entire beds of the participants were tilted at a 6° head‐down angle, and all the activities of the participants were performed in this continuous supervised 6° head‐down position (pillows not permitted), including eating, sleeping, toileting, and general hygiene. During the baseline data collection and recovery periods, the participants could freely move within the ward. During recovery, reconditioning sessions were administered by physiotherapists. Daily water and energy intakes were standardized and controlled throughout the study. Female participants did not take oral contraceptives and their menstrual cycle was not controlled for. Additional details regarding the whole AGBRESA protocol are given in (Kramer et al., 2021).

All participants underwent a 60‐day head‐down tilt bed rest and were pseudo‐randomly assigned to three groups matched for sex, age, and weight. The control group (*n* = 8, 2 females) underwent only bed rest without any countermeasure; the continuous artificial gravity group (*n* = 8, 3 females) was exposed to artificial gravity via a short‐arm centrifuge (1 *g* at the heart and about 2 *g* at the feet) for 30 min per day during the bed rest period; the intermittent artificial gravity group (*n* = 8, 3 females) was also exposed to the same *g*‐levels 30 min per day during the bed rest period, but the 30 min was split into six blocks of 5 min, separated by 3‐min breaks (Kramer et al., 2021). The distribution of the population in three groups is related to the overall objective of the AGBRESA study, which was to determine the efficacy of 30‐min daily artificial gravity as a countermeasure to the adverse effects of inactivity. No effects of the tested countermeasures were found (Kramer et al., 2021). We therefore pooled data from all the groups, in agreement with previous reports that have already demonstrated no statistical effect of these countermeasures on any cardiac or skeletal variables (Eggelbusch et al., 2024; Hendrickse et al., 2022; Hoffmann et al., 2021; Rabineau et al., 2022). Anthropometric measures have previously been described (Eggelbusch et al., 2024).

### 2.3 Cardiac MRI and analysis of cardiac function

**FIGURE A2:**
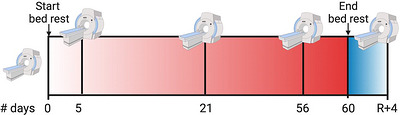
Study design. Magnetic resonance imaging was performed on the heart before (pre) after 5 (short‐), 21, and 56 (long‐term) days of bed rest, and after 4 days of reambulation (R+4).

MRI (3‐T Biograph, PET/MR, Siemens Healthineers, Erlangen, Germany) of the heart was performed 9 days before bed rest (0 days of bed rest), on days 5, 21, and 56, and after 4 days of recovery (R+4; Appendix Figure A2; Hoffmann et al., 2021; Rabineau et al., 2022). PET scans were not performed. Two‐, three‐, and four‐chamber long‐axis CINE movies (1.6 × 1.6 × 6.0 mm; echo time (TE): 1.43 ms, repetition time (TR): 39.24 ms, 25 phases) and a complete short‐axis CINE stack (1.6 × 1.6 × 7.0 mm^3^; TE: 1.43 ms, TR: 45.78 ms, 25 phases) with retrospective electrocardiogram gating were obtained from every participant at every time point. All MRI experiments were performed in horizontal (0°) position. Images were analysed using MEDIS software (Leiden, The Netherlands). LV end‐diastolic and end‐systolic volumes were used to calculate ejection fraction as a measure for systolic function. Maximal systolic ejection rate (*S* *′*) and diastolic function (ratio of the early or elastic (*E* *′*) to the atrial (*A* *′*) filling rates) were measured based on the volume‐time curves calculated with the long‐axes CINE movies. Data from one male participant could not be used due to technical difficulties.

### 2.4 LV haemodynamic forces

**FIGURE 1:**
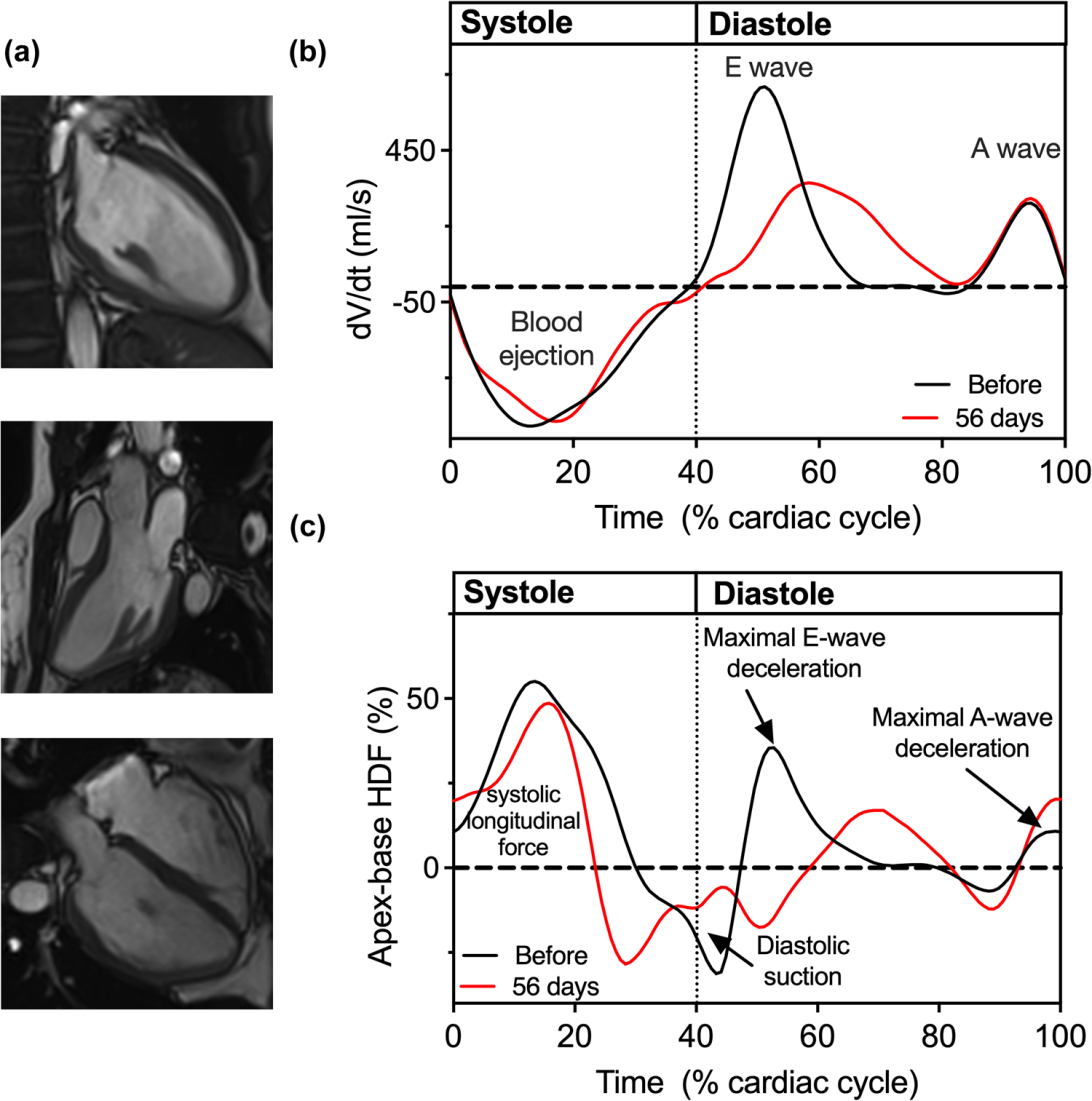
Assessment of intraventricular haemodynamic forces. (a) From top to bottom, representative examples of 2‐, 3‐, and 4‐chamber cine images of a human heart. (b, c) Representative examples of a participant before and after 56 days of bed rest. The changes in endocardial blood volume in the left ventricle over the cardiac cycle (*d* *V* */d* *t* in (b)), together with absolute volumes and gravity, is used to calculate the haemodynamic forces (HDF) curve in the apex–base direction in (c). The dotted vertical line represents the end of the systole. The systolic longitudinal force is the area under the HDF curve during the positive phase during the systole, while the diastolic suction is the area under the negative part of the HDF curve during the end of the systole and early diastole. The maximal E‐ and A‐wave decelerations are the maximal early and late diastolic peaks, respectively. The root mean square of this curve over the whole cardiac cycle is used as a quantification of the HDF signal.

LV haemodynamic forces were computed from the combination of the two‐, three‐, and four‐chamber long axis data sets, using dedicated software (Qmass v8.1.48.14 and QStrain v2.0.48.8, Medis, Leiden, the Netherlands). The endocardial borders were outlined in all frames of the cardiac cycle, and a time‐resolved 3D LV endocardial surface was subsequently reconstructed. The integral form of the conservation of linear momentum was applied to the LV cavity volume to provide a 3D haemodynamic force vector, representing the net force required to accelerate the blood mass within the cavity. This 3D haemodynamic force vector was then decomposed into components in the inferolateral–anteroseptal and apex–base directions. The forces were normalized to instantaneous LV cavity volume (to account for temporal and inter‐individual differences in cardiac size) and expressed as a percentage of the gravity acceleration constant to obtain a dimensionless measure (Lapinskas et al., 2019; Vallelonga et al., 2021). These measures represent imaging‐based indices of intracavitary blood acceleration and deceleration normalized to ventricular volume, reflecting flow–wall interaction and pump mechanics rather than direct measurements of myocardial force or intrinsic contractility. Conceptually, a similar wall motion in combination with a larger ventricular volume would indicate a larger change in integrated momentum of blood, and hence greater haemodynamic forces. The mathematical model for the haemodynamic forces was previously validated successfully against 4D flow MRI (Pedrizzetti et al., 2017). The vectorial angle of the haemodynamic forces was calculated relative to the orientation of the apex–base of the left ventricle during the complete cardiac cycle, and quantified during the systole. The quantification of the haemodynamic forces included the computation of the dimensionless root mean square of haemodynamic force over the entire cardiac cycle, the area under the systolic ejection and diastolic suction forces, as well as the maximal early and late diastolic deceleration forces. Figure 1 illustrates the analysis process and provides an example of a haemodynamic force curve.

### 2.5 Inward displacement

**FIGURE 2:**
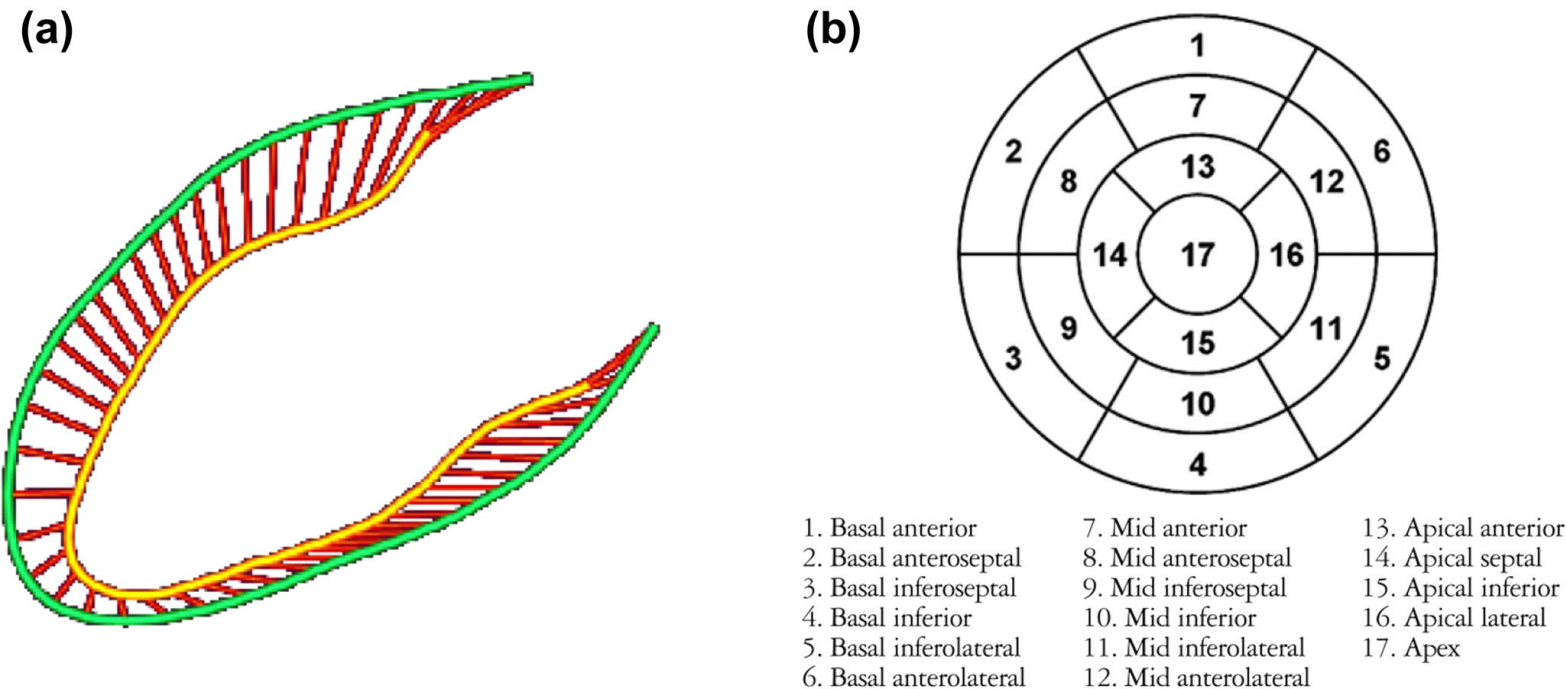
Inward displacement analysis of regional ventricular wall motion during bed rest in humans. (a) Regional inward displacement was assessed from 2‐, 3‐, and 4‐chamber cine images by assessing the movement of the endocardial wall in comparison with the ventricular midline. The green line represents the endocardial wall at end‐diastole, the yellow line the endocardial wall at end‐systole, with the red lines representing the endocardial wall motion. (b) The left ventricular cardiac wall was divided into 17 sectors, and regional displacement was assessed for each individual segment.

The analysis of the inward displacement was performed as described before (de la Pena‐Almaguer et al., 2021; Hegeman et al., 2023a, 2023b; Nabeta et al., 2023). Automated endocardial border detection with manual corrections was conducted for the two‐, three‐, and four‐chamber long axis images. Subsequently, the inward displacement feature was evaluated using the QStrain application (QStrain v2.0.48.8, Medis). The inward displacement of the cardiac wall is defined as the percentage inward motion of the wall toward the LV centre of contraction, independently from neighbouring segments (Figure 2a), and the LV wall was divided into 17 regional sectors (Figure 2b), from base to apex. We determined the inward displacement of the endocardial wall toward the true LV centre of contraction, from end‐diastole (last phase before a decrease in volume was detected) to end‐systole (last phase before an increase in volume was detected). Regional inward displacement was measured in the 17 standard LV segments across three levels (base, mid‐cavity, and apex), and expressed as a percentage of the total distance from the endocardial wall at the end‐diastole to the centre of the LV (Figure 2).

### 2.6 Plasma volume

Total plasma volume and total blood volume were measured using the optimized carbon monoxide (CO) rebreathing method before and after 5, 21, and 56 days of bed rest, as previously described (Horeau et al., 2022).

### 2.7 Statistical analysis

To identify statistical differences between time points, first a Shapiro–Wilks normality test was performed and, if data were normally distributed, a repeated ANOVA with Dunnett's *post hoc* analysis was performed for the bed rest period (baseline, day 5, 21, and 56). Student's paired *t*‐test was performed for 56 days vs. recovery, and recovery vs. baseline. Exploratory paired *t*‐tests (at baseline) and ANOVA analyses (for different responses) were performed between male and female participants. The effect of the anti‐gravitational intervention was tested with a repeated ANOVA. To evaluate the correlation between intra‐individual changes in some features, the repeated measures correlation coefficient *r*_rm_ (R library rmcorr) was used. Statistical analyses were considered significant if *P* ≤ 0.05. All data are presented as median boxplots with 10–90% percentiles.

## 3 RESULTS

**FIGURE A3:**
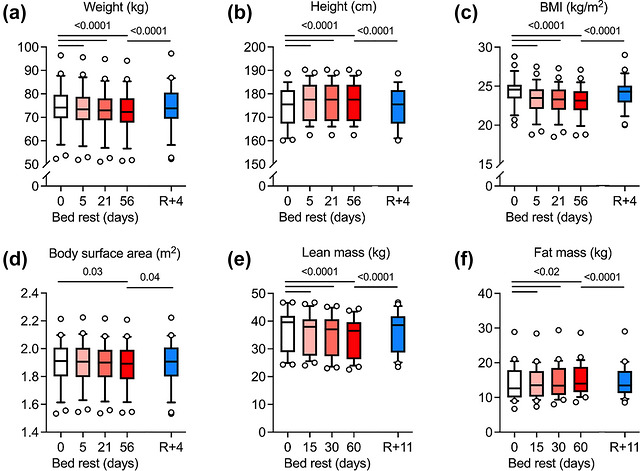
Anthropometric determinants during bed rest. (a–c) While body weight decreased throughout the bedrest, height increased (due to expansion of inter‐vertebrae discs), and body mass index (BMI) decreased significantly. Four days of reambulation increased body weight, decreased height, and increased BMI to pre‐bed rest values. (d) Body surface area only marginally decreased after 56 days of bed rest, but rapidly increased after reambulation. (e, f) Lean mass (trunk and extremities) significantly decreased, while fat mass transiently increased during the bed rest period. Upon reambulation, lean mass increased and fat mass decreased, but not to pre‐bed rest values. Box‐whisker plots with 10–90% percentile, *n* = 24, repeated ANOVA with Dunnet *post hoc* correction (bed rest) or paired *t*‐test (0/56/60 days vs. R+4/11).

Anthropometric determinants of the study participants during the bed rest period are provided in Appendix Figure A3.

### 3.1 Conventional measurements of cardiac function upon bed rest

**FIGURE A4:**
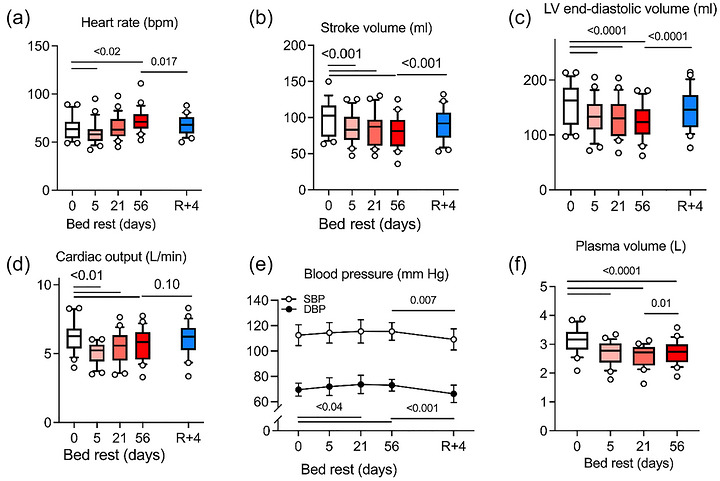
Cardiovascular alterations during bed rest. (a–d) While resting heart rate, measured during the MRI scan, decreased during short‐term bed rest (5 days), it gradually increased towards the 56 days of bed rest. Stroke volume, left‐ventricular (LV) end‐diastolic volume, and cardiac output rapidly decreased to a new steady‐state upon bed rest. Four days of recovery (R+4) decreased heart rate, and increased stroke and end‐diastolic volumes, while cardiac output tended to increase. (e) Systolic blood pressure did not change with bed rest, but diastolic blood pressure increased after 21 and 56 days. Recovery decreased systolic and diastolic blood pressures. (f) Plasma volume was only measured during bed rest, and decreased rapidly until 21 days, after which it significantly increased. Box–whisker plots with 10–90% percentile, or means ± SD (e), *n* = 24, repeated ANOVA with Dunnet *post hoc* correction (bed rest) or paired *t*‐test (56 days vs. R+4).

There was no significant difference in heart rate between baseline and day 5, but there was a slight increase from day 5 to day 56 (64 ± 12 bpm *vs*. 71 ± 13 bpm, *P *= 0.028). Heart rate significantly decreased after 4 days of reambulation (to 68 ± 10 bpm, *P *= 0.017 *vs*. day 56; Appendix Figure A4). Diastolic blood pressure gradually increased during bed rest (Appendix Figure A4). After 5 days of bed rest, LV end‐diastolic volume and stroke volume decreased from baseline values of 154 ± 37 and 99 ± 24 mL to 135 ± 34 and 86 ± 22 mL, respectively (both *P *< 0.0001; Appendix Figure A4). Until 56 days of bed rest, LV end‐diastolic volume continued to decrease to 125 ± 32 mL (*P *= 0.002 vs. day 5; Appendix Figure A4). However, LV ejection fraction remained unchanged from baseline (64 ± 4%) to day 56 (65 ± 4%, *P *= 0.95). During the recovery phase, ejection fraction slightly decreased to 61 ± 5% compared to day 56 (*P *= 0.016). Plasma volume decreased after 5 and 21 days, but marginally increased after 56 days (Appendix Figure A4f). Plasma volume was not assessed during the recovery phase.

### 3.2 Left ventricle haemodynamic forces

**FIGURE 3:**
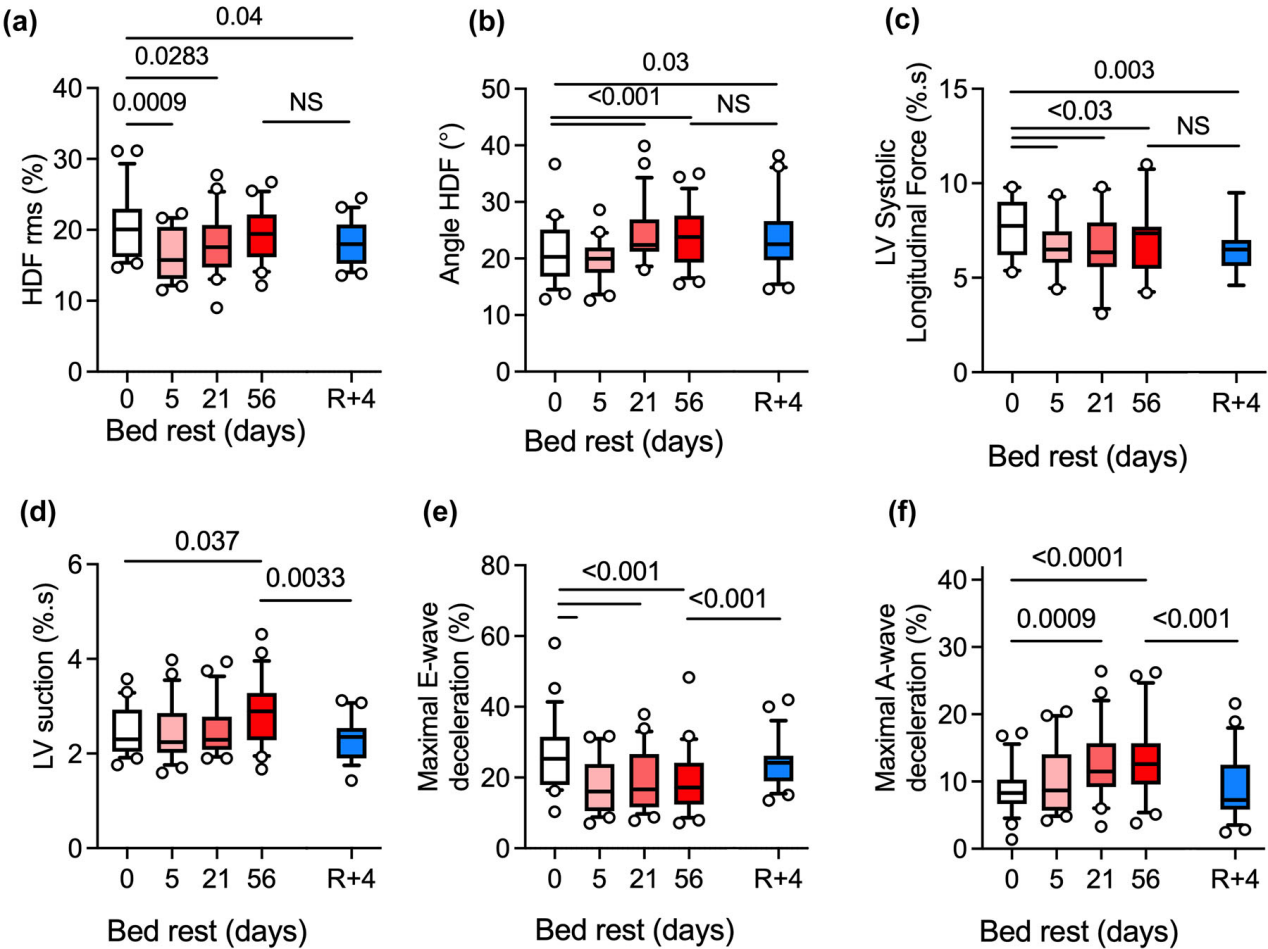
Longitudinal assessment of intraventricular haemodynamic forces in the apex–base direction, before, during, and after bed rest. (a) The root mean square (rms) of HDF significantly decreases during the early phases of bed rest and does not recover after 4 days of recovery (R+4). (b) An increased angle of the force vector relative to the apex–base axis during the systole suggests suboptimal blood flow dynamics after bed rest, which does not recover 4 days after bed rest. (c, d) After breaking the HDF signal up in specific periods during the cardiac cycle, the LV systolic longitudinal force significantly decreased upon bed rest (c), but the early diastolic suction significantly increased throughout the bed rest (d). (e) Maximal E‐wave deceleration was already reduced after day 5, indicative of an impaired relaxation of the heart. (f) The higher bed rest‐induced maximal peak during the atrial kick is indicative of a compensatory mechanism to maintain diastolic filling during long‐term bed rest. Data presented as median boxplots with 10–90% percentiles. Repeated ANOVA with Dunnett *post hoc* analysis or paired *t*‐test (0/56 vs. R+4), *n* = 24. HDF, haemodynamic forces; LV, left ventricle; R+4, 4 days of recovery.

**FIGURE A5:**
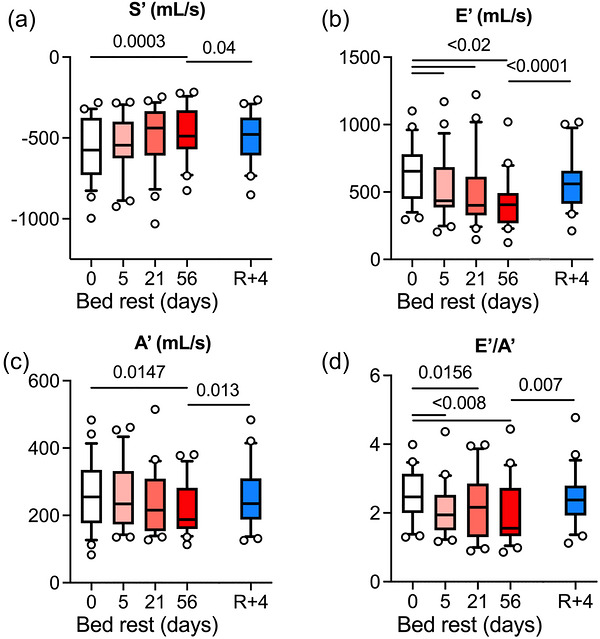
Bed rest reduces peak ejection (*S* *′*), and filling (*E* *′* and *A* *′*) rates, assessed via conventional volumetric analysis. Data presented as median box‐plots with 10–90% percentiles. Repeated ANOVA with Dunnett *post hoc* analysis or paired *t*‐test (0/56 vs. R+4), *n* = 24. HDF, haemodynamic forces; R+4, 4 days of recovery.

**FIGURE A6:**
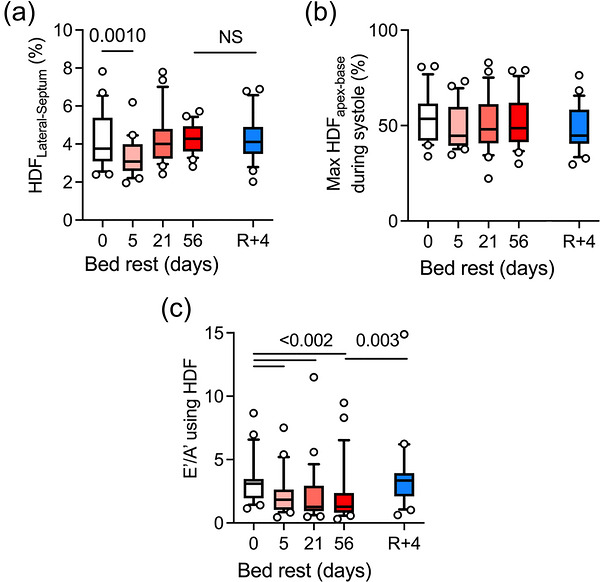
Additional measurements of haemodynamic force analysis and cardiac function. (a) The root mean square of the haemodynamic forces in the lateral–septal direction only changed upon 5 days. (b) The maximal apex–base haemodynamic force measured during the systole did not change with bed rest or recovery. (c) The ratio between the early and late diastolic deceleration using the apex–base data in Figure 3e, f rapidly decreased with short‐ and long‐term bed rest and recovered after recovery. Data presented as median box‐plots with 10–90% percentiles. Repeated ANOVA with Dunnett *post hoc* analysis or paired *t*‐test (0/56 vs. R+4), *n* = 24. HDF, haemodynamic forces; R+4, 4 days of recovery.

LV haemodynamic forces were quantified from standard two‐, three‐, and four‐chamber long‐axis cine images. Representative examples of LV images and computed haemodynamic forces are depicted in Figure 1. The root mean square of haemodynamic forces in the apex–base direction was reduced throughout the first 3 weeks of bed rest (−16 ± 16% and −10 ± 18% after 5 and 21 days, respectively, *P *< 0.0283), but recovered after day 56 (Figure 3). The haemodynamic forces in the inferolateral–anteroseptal direction were acutely reduced (−18 ± 23%) after day 5, but recovered to baseline values from day 21 onwards (Appendix Figure A6a). The vectorial angle of the haemodynamic forces (relative to the orientation of the apex–base of the left ventricle) was not altered after short‐term bed rest, but increased from 21 days bed rest (+20 ± 32% and +16 ± 32% after 21 and 56 days, respectively, *P *< 0.001; Figure 3b). There was a small but significant negative correlation between end diastolic volume and the vectorial angle of the haemodynamic forces before the bed rest and up to 5 days into the bed rest (*r*_rm_^2^ = 0.21, *P *= 0.03), which disappeared at later time points and during the recovery phase. The LV systolic ejection force (area under the haemodynamic forces curve during systole) was significantly lower throughout the whole bed rest period (−13 ± 14%, −13 ± 18%, and −10 ± 15% after 5, 21, and 56 days, respectively, *P *< 0.03; Figure 3c). Peak systolic haemodynamic force was not different across all time points (Appendix Figure A6b), while *S* *′* decreased only after day 56 (Appendix Figure A5a). These different markers for cardiac function did not rapidly recover to pre‐bed rest values after 4 days of recovery (Figure 3a–c). The absolute ejection fraction (ranging from 55% to 70%) correlated positively with the absolute root mean square of haemodynamic forces in the apex–base direction across all time points (*r*_rm_^2^ = 0.16, *P *= 0.001). The temporal changes in both variables were only marginally positively correlated between the baseline and bed rest time points (*r*_rm_^2^ = 0.05, *P *= 0.03), while they changed in opposite directions during the reambulation.

LV diastolic suction, which occurs during the very first phase of diastole, only started to significantly increase at day 56 of the bed rest period (by 15%; Figure 3d), possibly to maintain diastolic filling. On the other hand, the maximal diastolic deceleration, which occurs during the E‐wave, decreased significantly during both short‐term and long‐term bed rest (by 30–35%; Figure 3e, in accordance with a lower *E* *′*; Appendix Figure A5; Hoffmann et al., 2021). *A* *′* only significantly decreased after day 56 (Appendix Figure A5), but there was a significant increase in the maximal value for the late diastolic deceleration phase after day 21 and 56 (Figure 3f). The equivalent *E*/*A* ratio (the maximal early/late deceleration rate) using the haemodynamic force method (Appendix Figure A6c) reduced 27–30% upon short‐ and long‐term bed rest. The intra‐individual changes in this equivalent *E*/*A* ratio obtained using the haemodynamic force method correlated only slightly with the ones of volumetric *E* *′*/*A* *′* (*r*_rm_^2^ = 0.16, *P *< 0.001). These bed rest‐induced changes in diastolic haemodynamic forces returned to pre‐bed rest levels after 4 days of recovery (Figure 3 and Appendix Figure A6c).

**FIGURE A7:**
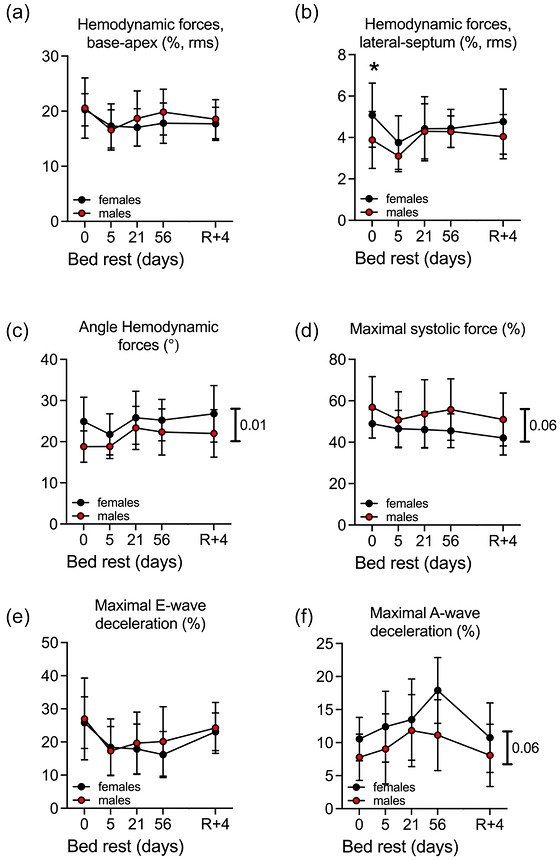
Sex differences in haemodynamic forces. (a, b) The changes in haemodynamic forces in the apex–base (a) and lateral‐septum direction (b) during bed rest was not different in males (*n* = 15) compared to females (*n* = 8), apart from a higher value for the haemodynamic forces in the lateral‐septum direction in females (b). (c) Females had a significantly higher angle of the haemodynamic forces, relative to the apex–base orientation, related to a less efficient outflow of blood. (d) Maximal systolic force tended to be lower in females. (e, f) No sex‐related differences were observed in the maximal E‐wave deceleration (e), but the maximal A‐wave deceleration tended to be higher in females (f). For clarity, the bed‐rest effects were not significant. Data are plotted as means ± SD. ANOVA repeated measures.

**FIGURE A8:**
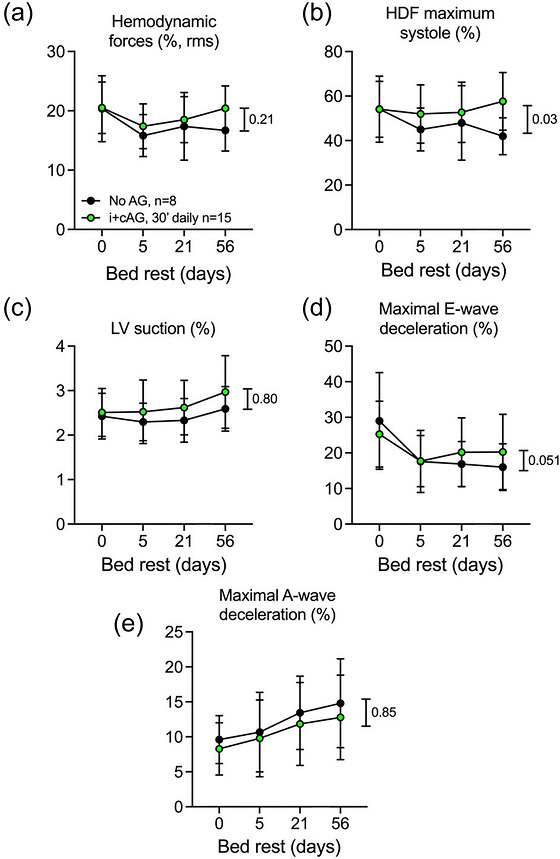
Artificial centrifugation marginally mitigates changes in haemodynamic forces (HDF). (a) Daily centrifugation (either continuous 30 min (cAG) or intermittent 6 × 5min (iAG), both interventions were combined) did not significantly change the time course of the strength of the haemodynamic forces. (b) A significant interaction effect for intervention × bed rest was found for maximal HDF during the systole, where the intervention group did not show a bed rest‐induced decrease. (c–e) The time course of left ventricular (LV) suction (c), maximal E‐wave deceleration (d), and maximal A‐wave deceleration (e) did not differ between intervention groups. A tendency of an interaction effect was observed for maximal E‐wave deceleration. Data shown as means ± SD. ANOVA repeated measures, *n* = 8 for no AG and *n* = 15 for cAG and iAG.

We performed some exploratory analyses to determine sex‐related difference in haemodynamic forces, although group sizes were relatively low. We did not observe sex‐related differences in the haemodynamic forces in the apex–base direction, but the angle of the haemodynamic forces and the haemodynamic forces in the lateral‐septum direction was higher in females at baseline (Appendix Figure A7). The artificial gravity intervention did not lead to major changes in haemodynamic force parameters (Appendix Figure A8).

### 3.3 Regional inward displacement of the cardiac wall

**FIGURE 4:**
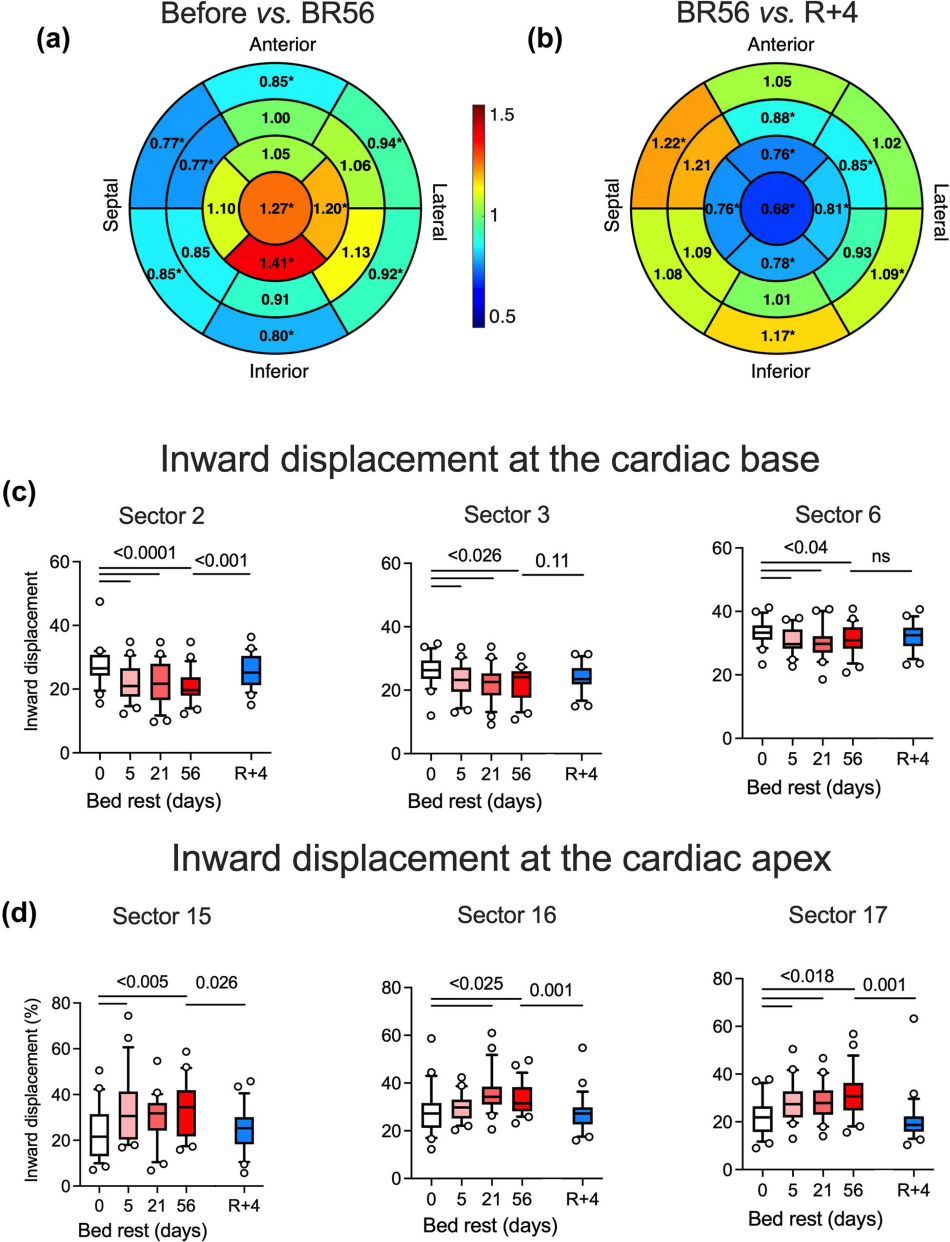
Inward displacement analysis reveals regional changes in ventricular wall motion during bed rest in humans. (a‐b): The relative changes in inward displacement after 56 days of bed rest (a) and after 4 days of reambulation (b) in all cardiac sectors. The wall motion of the cardiac base was significantly lower, while the apex showed hypercontractility upon 56 days of bed rest. (c‐d): The inward displacement of the wall motion (in %) in individual sectors of the base (c) and apex (d) regions show spatio‐temporal adaptations of the cardiac contractility upon bed rest in humans. Data presented as median box‐plots with 10%–90% percentiles. Repeated ANOVA with Dunnett *post hoc* analysis (0–56) or paired *t*‐test (56 vs. R+4), *n* = 24. * in A+B: *P *< 0.05. BR: bed rest; R+4: 4 days of recovery.

The wall motion was determined for each section independently, and relative changes after day 56 are displayed in Figure 4. We observed a disparity in the changes in cardiac wall contractility throughout the bed rest period. The motion of the cardiac base during the cardiac contractile phase was rapidly reduced upon 5 days of bed rest and remained lower up to day 56 (Figure 4a, c). We observed, however, a significant increase in apical relative motion after 5−21 days of bed rest (Figure 4a, d), indicative of an apical hypercontractility that became apparent at a later time point of the bed rest compared to the reduced cardiac base movement. The changes in LV size could only partially explain these opposite regional adaptations. Indeed, the intra‐individual changes in LV end‐diastolic volume were positively correlated with the ones of inward displacement in some basal segments (up to *r*_rm_^2^ = 0.31, *P *< 0.001, for segment 2), while they were negatively correlated with the changes of inward displacement in some apical segments (up to *r*_rm_^2^ = 0.18, *P *< 0.001, for segment 17). During the recovery period (4 days after the end of the bed rest period), inward displacement of most (but not all) cardiac segments returned to pre‐bed rest values (Figure 4b–d), suggesting a rapid readjustment of inward displacement and regional wall contractility upon recovery from bed rest.

## 4 DISCUSSION

This study used two innovative non‐invasive imaging parameters to evaluate changes in intraventricular pressure gradients and cardiac contractility during short‐ and long‐term head‐down bed rest in humans. Cardiac alterations have been previously described after bed rest and extended physical inactivity, though not leading to clinically apparent heart failure (Dorfman et al., 2008; Perhonen et al., 2001). Bed rest typically results in a reduction in LV size and atrophy, which, together with a smaller plasma volume and stroke volume, contribute to orthostatic intolerance observed after bed rest (Levine et al., 1997; Perhonen et al., 2001). Our longitudinal assessment demonstrated subclinical and partly transient alterations in haemodynamic forces throughout the cardiac cycle that cannot be determined by traditional cardiac assessments. Parameters related to diastolic alterations during early diastole rapidly changed, both at the beginning of the bed rest period and after 4 days of recovery, likely due to a readjustment of blood volume and preload (Caiani et al., 2014), or rapidly altered regional cardiac contractility. We also show that 2 months of bed rest in healthy people caused region‐dependent changes of the inward displacement at the cardiac base and at the apex. The increased apical relative motion during physical inactivity causes an increased LV suction, and together with the increased haemodynamic force induced by atrial contraction (i.e., A‐wave deceleration), ensures an appropriate cardiac filling during periods of physical inactivity.

### 4.1 Systolic alterations

The bed rest‐induced reduction in the root mean square of the apex–base haemodynamic forces was caused by changes happening at different periods of the cardiac cycle. Although peak systolic haemodynamic force remained unchanged, the area under the haemodynamic forces curve during systole and the maximal blood ejection rate *S* *′* were both lower during bed rest, similar to previous observations (Rabineau et al., 2022). A shorter LV ejection time (due to higher resting heart rate), a higher diastolic pressure, increasing the afterload, and a lower plasma volume, impairing LV filling, all likely contribute to both the reduced stroke volume and area under the haemodynamic forces curve during systole (Orter et al., 2022).

In parallel, as also shown in microgravity studies (Tordeur, 2025), bed rest reduced LV volume and promoted a more spherical ventricular geometry, which directly impacts intraventricular force generation. According to Laplace's law, wall stress scales with ventricular pressure and radius, and inversely with wall thickness. The reduced ventricular radius would lower global wall stress and the force available to accelerate blood, consistent with the observed reduction in systolic haemodynamic forces. However, haemodynamic forces are not solely determined by global pressure generation, but also by the spatio‐temporal pattern of myocardial contraction that governs intraventricular pressure gradients and flow acceleration.

This is reflected in the apparent discrepancy between a preserved peak systolic haemodynamic force and a lower peak ejection velocity, which can be explained by a decreased efficiency of the cardiac contraction, as bed rest increased the angle of haemodynamic forces relative to the optimal apex–base direction. Before the bed rest, the angle of the haemodynamic forces was associated with the size of the end‐diastolic volume, but the changes in this angle at later time points were not associated with those of end‐diastolic volume per se. The geometry of the LV contraction, measured with the regional inward displacements of the cardiac wall, was altered as a consequence of bed rest. A more spherical left ventricle explains why few changes of haemodynamic forces were observed in the inferolateral–anteroseptal direction, while the contractility decreased in the basal segments. The bed rest‐induced reduction in relative motion at the base of the left ventricle contrasted with an increased inward displacement at the apex. A smaller LV volume could only partially explain these regional differences, as underlined by the low values of the associated repeated‐measures correlation coefficients. In previous studies, the fluid shift caused by acute changes in position from standing to supine led to a more pronounced increase of long‐axis shortening than of short‐axis shortening, but also a larger increase in the lateral than in the septal part (Orter et al., 2022), something that we observed here (Figure 4). As such, changes in volumes, orientation, shape (Summers et al., 2010), and mass (Summers et al., 2005) of the left ventricle each play a role in the altered systolic function following bed rest. Together, these findings indicate that changes in LV volume, geometry, and regional contractile patterns jointly impair the efficiency with which myocardial work is translated into directed intraventricular flow, linking local wall mechanics to global haemodynamic force generation.

It is noteworthy that the decrease observed following bed rest in the root mean square of the apex–base haemodynamic forces, as well as in its systolic area under the curve, remain subclinical. Even at the end of the bed rest period, the values of these parameters remained above those typically found in older heart failure patients with mid‐range and reduced ejection fractions (Lapinskas et al., 2019). The overall root mean square of the haemodynamic forces correlated to ejection fraction in patients with heart failure with preserved ejection fraction (Arvidsson et al., 2022) and in our participants, but changes in overall haemodynamic forces did not strongly correlate to changes in ejection fraction, particularly as ejection fraction decreased and haemodynamic forces increased at the reambulation phase. As such, we conclude that the cardiac physiology underlying changes in haemodynamic forces are distinct from ejection fraction only.

### 4.2 Diastolic alterations

The early passive filling was negatively affected by short‐term bed rest, as we observed a reduction in *E* *′* and maximal E‐wave deceleration after only 5 days of bed rest. In parallel, an increase in the maximal A‐wave deceleration was observed after day 21, which slightly compensated for the decrease of passive filling. Such a situation logically led to a decrease of the *E* *′*/*A* *′* ratio, as assessed by volumetric measurements as well as our haemodynamic force assessment. However, these two methods of evaluating the relative contribution of passive and active filling are not equivalent, as highlighted by the low value of the correlation coefficient between their intra‐individual changes. Similar alterations of the diastolic function, including a reduced dynamic ventricular filling, have repeatedly been reported in response to bed rest (Caiani et al., 2014; Hastings et al., 2012; Hoffmann et al., 2021; Levine et al., 1997; Perhonen et al., 2001; Rabineau et al., 2022).

Most of the diastolic changes are likely a consequence of changes in blood volume and preload rather than intrinsic (structural) ventricular relaxation properties (Caiani et al., 2014; Hastings et al., 2012; Levine et al., 1997). Previous work found a bed rest‐induced reduction in maximal untwisting rates (Dorfman et al., 2008) together with a longer isovolumic relaxation time (Carrick‐Ranson et al., 2013). However, active relaxation was not altered during volume infusion, highlighting the role of preload on the features traditionally used to evaluate the early diastolic filling phase (Carrick‐Ranson et al., 2012). Here, diastolic suction derived from our haemodynamic forces analysis was normalized by the instantaneous volume of the left ventricle and, since it corresponds to an area under the curve, it could account for a longer isovolumic relaxation time. The fact that the markers of diastolic function rapidly recovered upon 4 days of reambulation is in line with an expected rapid recovery in plasma volume. Further studies with experimental correction of the bed rest‐induced reduction in preload are, however, required to fully understand the altered haemodynamic forces during diastolic suction.

### 4.3 Non‐invasive cardiac imaging parameters

New imaging parameters such as haemodynamic force or inward displacement analysis allow for the longitudinal detection of sub‐clinical changes in myocardial function which can help to understand the altered cardiac function upon various environmental and genetic stressors. Recent intervention data support the value of sensitive non‐invasive imaging for detecting subtle cardiac adaptations to altered activity exposure. In a 6‐month trial, less sedentary behaviour was associated with modest improvements in exercise LV strain, highlighting the ability of imaging‐derived markers to capture early or subclinical remodelling (Norha et al., 2026). While haemodynamic forces do not directly represent myocardial contractility, the analysis of haemodynamic forces to evaluate the relative contribution of active and passive filling has the potential to be more sensitive than the methods based on peak filling rates, such as *E* *′*/*A* *′*, where smaller relative changes are observed. Indeed, *A*′ was only observed to decrease relative to baseline values after day 56, while the maximal A‐wave deceleration increased already after day 21 of the trial.

The quantification of diastolic suction with haemodynamic forces includes the information of preload and isovolumic relaxation time. However, it is too early to conclude that this parameter is better than the others traditionally used to assess diastolic suction, as more studies are needed to evaluate its dependence on preload and potentially additional confounding factors.

### 4.4 Study limitations

One inherent study limitation was that the experiments inside the MRI scanner were conducted in the horizontal position, and not in the 6° head‐down position that was maintained during the rest of the bed rest period. It is unknown what effect this had on the outcomes of this study. Many physiological alterations in the cardiovascular system occur simultaneously during bed rest, including a decrease in plasma volume and an increase of heart rate. It is unclear if alterations in one of these parameters would independently affect haemodynamic forces and regional contractility of the left ventricle. Since we did not study intracellular adaptations of the heart to bed rest, it remains elusive whether structural alterations in cardiomyocyte size or metabolism contribute to the altered cardiac contractility upon bed rest. Similarly, the regional changes in cardiac wall motion likely have an intracellular component, but this is currently unknown. Future research on larger cohorts is required to provide further information on normal ranges for healthy individuals, including sex‐ and age‐related differences (Ferrara et al., 2021). Independent larger cohort clinical studies are required before these novel non‐invasive cardiac imaging parameters can also be used for clinical evaluation of early changes in cardiac function during the development of cardiovascular diseases.

### 4.5 Conclusion

In this study, we applied two novel non‐invasive imaging modalities to a longitudinal study of strict head‐down bed rest in humans to assess the evolution of LV haemodynamic forces and regional wall motion throughout the cardiac cycle. Our findings revealed that bed rest not only increased active diastolic filling but also induced regional hypercontractility at the apex, along with an increased LV suction during early diastole to ensure appropriate cardiac filling. On the systolic side, the distribution of the haemodynamic forces indicated an altered efficiency of the cardiac contraction, suggested by a larger angle of haemodynamic forces relative to the optimal apex–base direction. These novel spatio‐temporal assessments of cardiac function provide new insights into the longitudinal alterations that occur after bed rest‐induced physical inactivity.

### 4.6 Clinical perspectives

Non‐invasive imaging tools can be useful for studying longitudinal alterations in cardiac function upon environmental stressors. In this study, we used two such methods to examine how strict head‐down bed rest affects intraventricular haemodynamic forces and regional cardiac wall motion. Our findings reveal that haemodynamic force production decreases after long‐term bed rest, but suction during the early diastole increases, likely due to hypercontractility of the apex. These imaging techniques offer a unique way to gain novel insights into cardiac alterations that occur in response to environmental stressors.

## AUTHOR CONTRIBUTIONS

Pierre‐François Migeotte and Jens Tank conceived the idea and the design of the cardiac MRI protocols for AGBRESA. Pierre‐François Migeotte made cardiac MRI data available for analysis. Jérémy Rabineau and Fabian Hoffmann contributed to data acquisition. Fatimah Al‐Darwish, Bram F. Coolen, David Hautemann, Gustav J. Strijkers, and Rob CI Wüst performed (statistical) analyses. Rob CI Wüst and Jérémy Rabineau drafted the manuscript. All authors contributed to data interpretation and editing. All authors have read and approved the final version of this manuscript and agree to be accountable for all aspects of the work in ensuring that questions related to the accuracy or integrity of any part of the work are appropriately investigated and resolved. All persons designated as authors qualify for authorship, and all those who qualify for authorship are listed.

## CONFLICT OF INTEREST

D.H. was an employee of Medis at the time of the study. The other authors report no conflicts of interest with industry partners.

## Data Availability

Data are available upon reasonable request to the corresponding author (RCIW).
